# Tetanus

**Published:** 1916-06

**Authors:** 


**Tetanus.** Sir David Bruce, Lancet, No. 4808, 1915, Vol. 189, gives an analysis of 231 military cases treated in home hospitals. Incubation varied from 3 to 157 days. 37 received a prophylactic dose of serum, 19 dying—51.3%. 215 cases were given serum immediately after the development of symptoms, the mortality varying from 42.8% for a group treated intraspinally to 85.7% for a group treated intravenously. (Note: The groups evidently overlap so that it is impossible to present full statistics).

